# Exploring Acoustic Detection of α-Synuclein Fibrils

**DOI:** 10.1007/s10930-024-10241-w

**Published:** 2024-12-04

**Authors:** M. Brun-Cosme-Bruny, L. Gerfault, V. Mourier, N. Torres, P. Bleuet

**Affiliations:** 1 Univ. Grenoble Alpes, CEA, Leti, Clinatec, 38000 Grenoble, France; 2 Univ. Grenoble Alpes, CEA, Leti, 38000 Grenoble, France

**Keywords:** α-synuclein, Fibrils, Frequency, Spectrum, Ultrasounds

## Abstract

Over the past decades, the incidence of Parkinson’s disease (PD) cases has doubled in industrialized countries. While patients over 70 years old still represent more than half of the cases, the disease is increasingly affecting younger individuals. Environmental factors have been implicated, such as the effects of certain pesticides or chemicals on neurons, such as rotenone or 1-methyl-4-phenyl-1,2,3,6-tetrahydropyridine (MPTP). Researchers have also demonstrated the influence of genetic mutations in younger patients. A-synuclein is a protein encoded by the SNCA gene, known to undergo various mutations in hereditary cases of PD. These mutations alter the composition and spatial arrangements of α-synuclein. The proteins, originally of linear shape, aggregate during the progression of PD, forming fibrillary structures that propagate through brain tissues. Among the physical therapies investigated for treating α-synuclein aggregation, ultrasonic waves, capable of altering protein and cell behaviors, have recently been used to disrupt α-synuclein fibrils within tissues in cellular and animal models, with the hope of developing treatments based on ultrasound properties. However, detecting fibrils typically requires invasive and non-biocompatible chemical compounds or cumbersome machinery. In this study, our acoustic experimental setup allowed us to investigate the response of α-synuclein to ultrasound perturbations. By capturing the transmitted wave across proteins over a frequency range 10 kHz to 10 MHz, no ultrasound signature indicating the presence of proteins was observed.

**Significance Statement**: The results report there is no ultrasound signature of the presence of α-synuclein fibrils, from 10 kHz to 10 MHz.

## Introduction

Among the neurodegenerative diseases of the century, Parkinson’s disease (PD) is one of the most investigated. While the illness primarily affects patients over 70 years old, an increasing number of younger individuals are also being diagnosed. Some chemicals implicated have been identified, such as rotenone or MPTP. Recent studies have also determined genetic mutations as responsible for hereditary transmission of PD. For instance, mutations of PARK1 [1] and PARK2 [2] genes induce deterioration in protein regulation, and SNCA gene mutation causes changes in α-synuclein behavior [3]. This protein, involved in dopamine transport, is originally linear, but can aggregate sporadically, and mutations in its coding gene can alter its tendency to adopt secondary structures, making it more prone to oligomer formation, followed by fibril [4] and aggregate formation [5, 6]. Besides, post-translational modifications are common in animal cells and are a significant factor in the aggregation process [7]. Accumulation of aggregates increases oxidative species in neurons, thus accelerating the progression of PD [8]. As of today, pharmacological [9], gene [10], and stem cell therapies [11] appear to be promising treatments that have shown the potential to slow down the progression of the disease. Photobiomodulation is a physical therapy that has demonstrated in preclinical trials its efficacy in slowing down PD [12], by irradiating the *Substantia Nigra*, the part of the midbrain affected by the disease. This optical stimulation would reactivate the respiration process by mitochondria. A field of therapeutic investigations has emerged during the last decades targeting fibrils aggregates themselves [13, 14]. Among them, ultrasound stimulation appears to be less invasive since it does not require any additional chemicals. Insonifying fibrils around 20 kHz with certain intensity parameters breaks them down according to in vitro research [15]. Other strategies aimed at acoustically silencing gene expression of α-synuclein to prevent aggregation have been tested in preclinical assays on mice [16].

Despite the urgency to find adequate treatments, none existing is able to adapt the intensity of ultrasound stimulation to the progression of PD, in order to provide an on-demand therapy for each patient. Monitoring the state of the disease presently requires heavy and invasive processes, such as radioactive chemical injection for probing dopamine levels by PET scan [17], impossible to perform regularly enough to use it for on-demand treatment. Α-synuclein aggregates not only represent a specific biomarker of PD progression but can also be detected and quantified by optical reflection spectra (FTIR) [18]. However, its use remains in vitro as the equipment cannot be miniaturized for in vivo implantation and diagnosis. Other techniques such as Thioflavin T or Congo red staining can efficiently reveal α-synuclein fibrils [19, 20], but they require biopsies or lumbar puncture from patients and are not suitable for non-invasive and regular monitoring [21].

Ultrasounds are another physical mean for material detection, specifically for entities named metamaterials [22]. When exposed to ultrasounds, their particular spatial organization interacts with ambient fluid to generate modulation in the transmitted wave, at a defined frequency, either by absorption or by cavitation. Indeed, in the case of fibrils, numerical simulations have demonstrated their destruction at 20 kHz is caused by anticipated cavitation [15]. Oxygen bubbles precociously emerge near the fibrillary structure and oscillate, by a coupled interaction with surrounding water as the ultrasound waves pass by, before imploding. This collapse generates shockwaves strong enough to disrupt fibrils and aggregates.

While preclinical assays aim at a therapeutic objective [23–25], none has focused its research on how ultrasounds could detect and quantify fibrils. In this paper, we investigated how we could potentially use ultrasound to probe α-synuclein fibrils by analyzing spectra of transmitted waves.

## Material and Methods

### Production of α-Synuclein Protein

The SNCA gene sequence, which codes for α-synuclein, is part of the plasmid PT7-7 asyn WT obtained from Addgene (#36046). BL21(DE3) bacteria were cultured on LB (Luria–Bertani) agarose containing nutrients, then transformed with the plasmid and grown in 1 L of LB liquid culture. Bacteria were selected using ampicillin, and growth was halted during the exponential phase. IPTG (0.1 mM) was added to the culture to induce α-synuclein production for 5 h at 37 °C and 200 rpm. The bacteria were subsequently centrifuged at 4000 rpm at 4 °C. The pellet was washed in 30 mL of Tris–HCl buffer (30 mM, pH 7.2), then stored at −80 °C.

### Purification of α-Synculein

Cell lysis was carried out on ice, involving resuspension of the cell pellet in a 40 mM Tris acetate buffer, pH 8.3, containing 1 mM EDTA, 1 mm phenylmethylsulfonyl fluoride (PMSF), and ultrasonicated with an output power of 12 watts applied in 30-s pulses followed by a 30 s pause, totaling a 5-min ultrasonication time. Subsequently, cell debris were removed by centrifugation at 20,000 rpm in a JA-20 rotor (Beckman Coulter) for 20 min at 4 °C. The lysates were then filtered through 0.22 μm membranes and loaded onto a XK16/20 Q FF anion-exchange column (20 mL Sepharose Cytiva) connected to an Akta FPLC1 Pure system (GE Healthcare), equilibrated with 20 mM Tris–HCl, pH 8.0. A-synuclein was eluted at a flow rate of 2 mL/min by applying increasing concentrations of up to 1.0 M NaCl in 20 mM Tris, pH 8.0, using a linear gradient applied over 10 column volumes. A-synuclein eluted at approximately 300 mM NaCl. A-synuclein-enriched fractions (as determined by SDS-PAGE) were then pooled and further purified by gel-filtration chromatography using a HiLoad 16/600 Superdex 75 µg (Cytiva) equilibrated with 10 mM Tris, pH 7.4, 2.7 mM KCl, 137 mM NaCl. Proteins were eluted at 2 mL/min; pure fractions were combined and dialyzed against PBS 1X at 4 °C using a 7 kDa cutoff dialysis membrane. A UV–VIS spectrum (UV-1800 Shimadzu) of the final solution described an absorption peak at 277 nm showing, despite the absence of tryptophan in α-synuclein sequence, that the proteins were purified at 98%. The final concentration of 2.9 mg/mL was determined with a molar extinction coefficient of 5120 M^−1^ cm^−1^ and a molecular weight of 14,460 Da. The aliquots were then stored at − 80 °C.

### Fibrillation of α-Synuclein and Phantom Creation

Thioflavin T powder (#T3516 Merck) was reconstituted at 1 mg/mL in methanol, then aliquoted and stored at − 20 °C. Purified α-synuclein was thawed at room temperature and heated to 37 °C, while vortexed at 1000 rpm for 5 days. To assess the formation of α-synuclein fibrils, two parallel methods were used. On the one hand, for each incubation period (over 5 days), 20 µL of incubated solution was diluted in 80 µL of PBS, and stained with 0.65 µL of Thioflavin T. 5 replicates for each period were analyzed for standard deviation computation represented as error bars. A 450 nm wavelength were set for excitation and the emission wavelength was 485 nm, with a Tecan M1000 spectrophotometer (Gain 146, integration time 20 µs). On the other hand, for observation under a Transmission Electron Microscope (TEM) at day 5, 10 µL of the solution was diluted to 1/10th and incubated 5 min on a Formvar coated 200 mesh size coper grid. The sample was washed with Milli-Q water and stained with 1% sodium silicotungstate. Images were acquired with a Tecnai F20 (FEI) electron microscope operated at 80 kV, coupled to a Ceta FEI 4kx4k camera. The fibrils were subsequently diluted in PBS 1X with 1% agarose to a final monomer concentration of 300 µg/mL. The solution was then solidified in a cylindrical flask with a diameter of 2.5 cm and a height of 4 cm. Concurrently, control phantoms were prepared in the same manner without α-synuclein fibrils. In total, 5 α-synuclein phantoms and 5 control phantoms were examined.

### Experimental Acoustic SetUp

A water tank with dimensions (30 × 30 × 35 cm) was used to investigate transmitted waves through an agar phantom infused with α-synuclein fibrils. The experimental setup included a fixed-arm system comprising an acoustic transducer (Ultran #GS50-D13 for 10 to 100 kHz range, and Olympus #U8421005 for 0.5 kHz to 10 MHz range), a phantom for receiving and transmitting the waves, and a needle hydrophone (Precision Acoustic #NH1000). Successive pulses of 5 cycles each were applied over these frequency ranges, with a step of either 2 or 500 kHz, depending on the transducer used. They were amplified by an amplifier (Precision Acoustic #HP) and launched through the phantom. The transmitted signal captured by the hydrophone was then recorded on an oscilloscope and stored on attached computer. The entire acoustic system was driven using Python programming.

## Results

### Fibrillation of In Vitro α-Synuclein

Α-synuclein is a protein that can be obtained through bacterial production. Recombinant α-synuclein proteins from bacteria are not subjected to post-translational modifications like animal proteins are, which may lead to differences in their physico-chemical properties. These chemical modifications appear to have both up-regulating and down-regulating effects on fibril formation [7], as well suggesting that they are not crucial for aggregation. BL21(DE3) bacteria were transformed with SNCA DNA encoding for α-synuclein, cultivated in LB liquid medium, and induced to produce the protein using IPTG. Following bacterial membrane lysis by sonication, α-synuclein extraction and purification were carried out using an ion exchange column, followed by HPLC chromatography. The purified α-synuclein (> 98%) was then aliquoted (2 mL, 2 mg/mL) and frozen at − 80 °C.

**Fig. 1:**
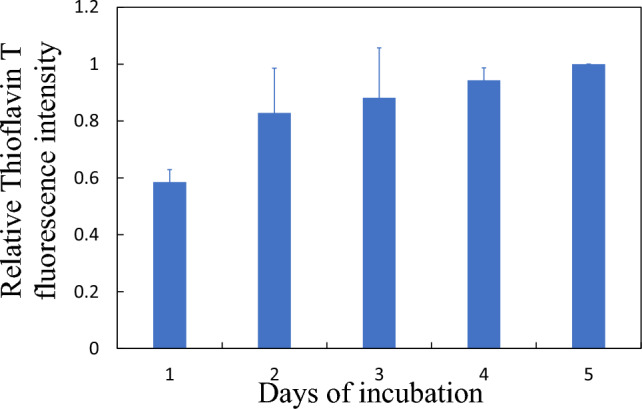
Histogram of the relative fluorescence intensity of Thioflavin T in PBS, a fibril fluomarker, against incubation duration, revealing when the fibrillation process stops by the presence of a plateau. Data are normalized by the maximum reached for 5 days incubation. Error bars represent standard deviations on 5 independent experiments of incubation

Fibrils were prepared by incubating and vortexing aliquots at 37 °C, 1000 rpm for 5 days. The process of fibrillation was monitored using Thioflavin T marker, a fluorophore that binds to amyloids of proteins like α-synuclein. Throughout the incubation period, 20 µL of α-synuclein solution was extracted daily for Thioflavin T analysis via a plate reader. Figure 1 shows the histogram illustrating the progression of fibrillation as revealed by fluorescence. Fibrillation increases day by day reaching an plateau about the 4th–5th days.

**Fig. 2:**
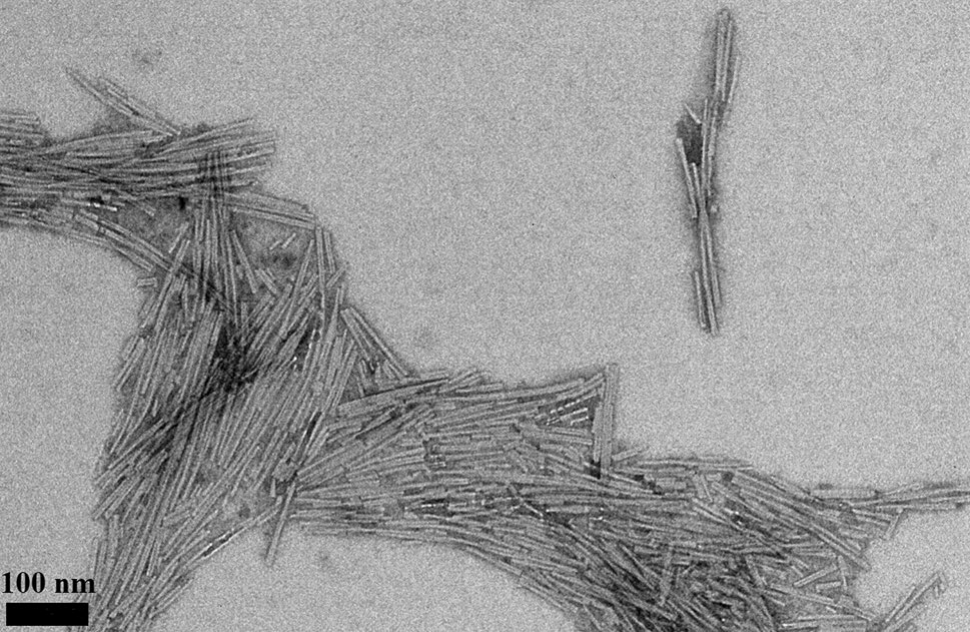
TEM image of fibrils aggregates

As a secondary confirmation of fibrillation and in order to validate the dimensions of the fibrils, we imaged the final solution after 5 days using transmission electron microscopy. Diluted fibrils (1/10th) were stained with sodium silicotungstate before being examined under the microscope. The image in Fig. 2 depicts magnified aggregates of fibrils that align with the mean length and diameter reported in the literature, approximately 1 µm long with a diameter of 10 nm. Background noise observed in the images could either be attributed to measurement noise or the presence of α-synuclein oligomers, which are precursors of fibrillation [26].

**Fig. 3:**
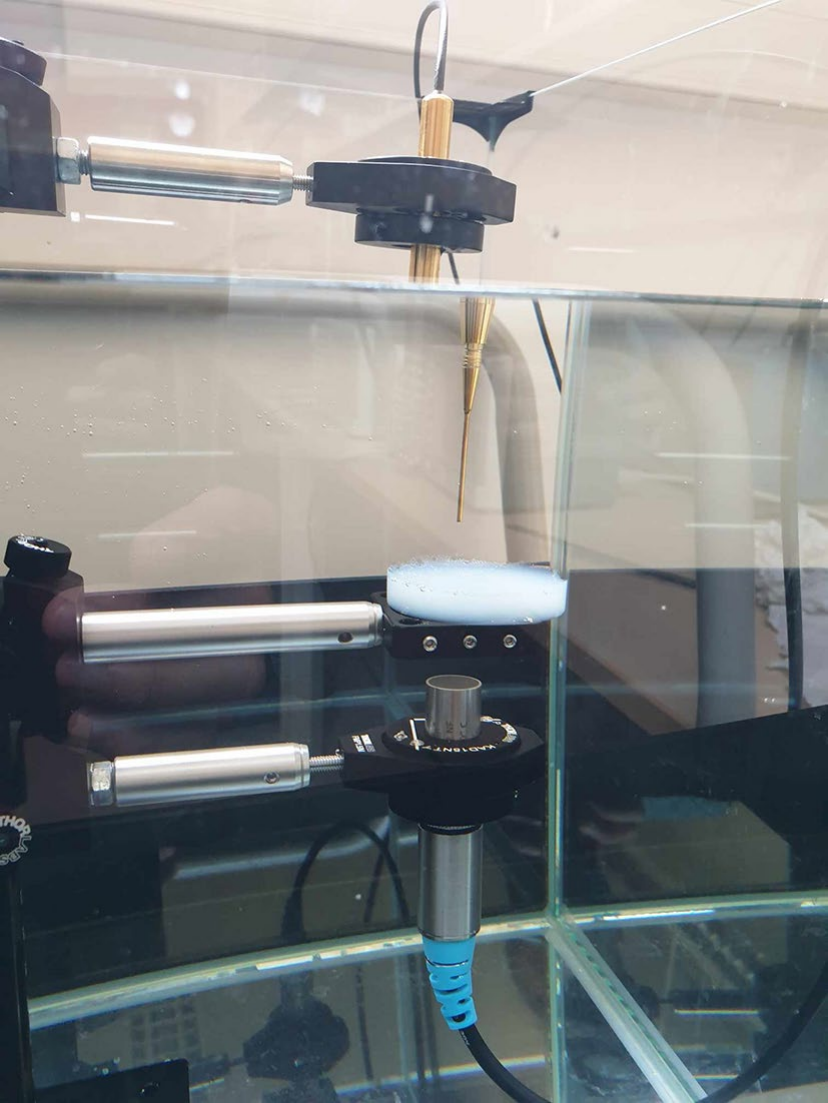
Photograph of the acoustic experimental setup. In the center, the agar phantom is doped with fibrils, receiving waves from the transducer (below) and transmitting it to the needle hydrophone (above)

Five phantoms consisting of agar doped with α-synuclein fibrils were tested and compared to five control phantoms (agar without fibrils), as well as to a water sample (absence of a phantom). To prepare the phantoms, we mixed 1% agarose in PBS 1X with α-synuclein fibrils at a monomeric concentration of 300 µg/mL (Fig. 3). It is worth noting that this concentration overrates the natural concentration found in live neurons (about 0.2 µg/mL [27]), as we intentionally overdoped the phantom to ensure detection without missing any signal.

### Acoustic Measurements

**Fig. 4:**
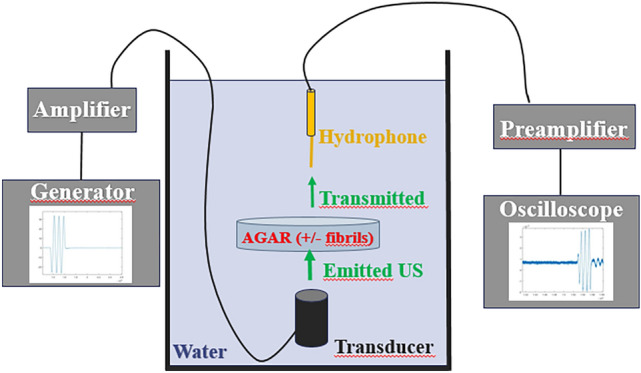
Schematic of the experimental setup. The transducer and hydrophone are immersed with the agar phantom in a water tank. The acoustic pulse signal is designed by a function generator and amplified before being converted into an acoustic wave by the transducer. After transmission through the agar phantom doped with fibrils (or not), the wave is converted back to an electric signal by the hydrophone and sent to an oscilloscope after being amplified for recording

Each phantom was positioned on a system of fixed arms within a water tank, as depicted in Figs. 3 and 4. Beneath the phantoms, a transducer emitted acoustic waves ranging from 10 to 50 kHz, or alternatively, from 0.5 to 10 MHz. Sinusoidal pulses of five cycles each were successively launched at specific frequencies within these ranges. These pulses passed through the agar phantom along the acoustic path before reaching a needle hydrophone, where acoustic signals were converted into electrical data and recorded on an oscilloscope. Both the transmitted signals were recorded and analyzed for comparison. The entire setup was automated using Python programming.

**Fig. 5:**
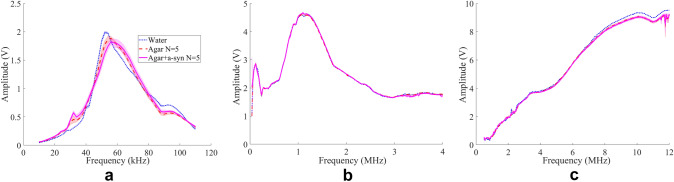
Spectra of the acoustic waves. Waves are transmitted through phantoms doped with fibrils (full line, Agar + α-syn), compared to spectra through phantoms without fibrils (dash line, Agar) and transmitted without phantom (dots, Water). Each graph corresponds to the acquisition with different transducers: highly sensitive at 60 kHz (**a**), at 1 MHz (**b**), and at 10 MHz (**c**). Shading represents standard deviation over 5 phantom replicates

For each pulse launched at a tabulated frequency, we measured the amplitude of the transmitted wave. This allowed us to generate Fig. 5, which illustrates the amplitude against frequency. Data are presented for two frequency ranges: 10 to 100 kHz and 100 kHz to 12 MHz. These measurements were obtained using three different transducers as described in Material and Methods section.

In each condition, the spectrum of transmitted waves exhibits a peak at a specific frequency due to the resonance of the transducer used. Comparing the control phantoms made of agar with water samples highlights the damping effect of agar itself on the transmitted waves, underscoring the importance of solely comparing control agar and α-synuclein agar phantoms. As a result, the spectra of the latter are nearly identical, indicating the absence of any discernible acoustic signature associated with the presence of α-synuclein fibrils.

## Discussion

In this study, we sought to determine whether the detection of fibrils could be achieved by ultrasound investigation. Our findings revealed no characteristic signature within the frequency ranges of 10 kHz–100 kHz and 100 kHz–12 MHz. This lack of distinction was observed between the waves transmitted through the control phantoms and those through the phantoms doped with α-synuclein fibrils. While our results were inconclusive, it is important to note that this exploratory study did not exhaust all possible parameters for highlighting the presence of fibrils.

Firstly, the frequency ranges explored, although wide, may have overlooked resonance frequencies in unexplored ranges. Utilizing other types of transducers capable of exploring frequencies beyond 10 MHz or below 10 kHz could provide valuable insights. However, the latter range falls within the audible domain and may not be relevant for our intended application in the brain. Additionally, variations in sound velocity within doped media at different doping densities could potentially reveal changes indicative of fibril presence [28].

Although cavitation appears chaotic for the detection process, exploring this phenomenon might yield fruitful results. Noise generated by bubble formation has shown promise in proximity to fibrils [29], suggesting that cavitation noise analysis could be used for fibril detection and quantification. However, the ultrasound frequencies required to produce cavitation are close to audible ranges (~ 20 kHz) and the intensity (> 3 kPa) may degrade surrounding tissues [30, 31].

The use of a fibril doping concentration of 300 µg/mL in agar phantoms was deemed excessive and not physiologically relevant. While the true concentration of fibrils in brain tissues remains unclear, researchers estimate α-synuclein concentration to be in the range of µg/mL. It is important to note that this value does not discriminate between linear, fibrillary, or oligomeric conformations, suggesting that the real concentration of fibrils is likely much lower. Future research endeavors will strive for more realistic and refined models.

Furthermore, our experiments solely focused on transmitted waves. However, for surgical applications, the device will eventually need to acquire echoes of reflected waves. This requires a transducer capable of both emitting and receiving waves, which is more practical for implanting medical devices. Once a resonance frequency for fibrils is identified, packaging medical devices based on acoustics will likely utilize CMUT or PMUT technologies [32], which feature highly sensitive micro capacitive or piezoelectric membranes respectively.

Finally, concerning ultrasound therapy, fibril disruption has been shown to generate new aggregation nuclei, therefore increasing the final number of them [33]. Sonication used in for treatment will thus generate new nuclei to seed for fibril formation. Unless relying on lifelong sonication therapy for the patient, it will be necessary to find a complementary physical or chemical therapy capable of destroying the nuclei.

## Data Availability

Data are available in the following link: https://data.mendeley.com/datasets/nbcxcgbgvv/1.
